# A New Scene Sensing Model Based on Multi-Source Data from Smartphones

**DOI:** 10.3390/s24206669

**Published:** 2024-10-16

**Authors:** Zhenke Ding, Zhongliang Deng, Enwen Hu, Bingxun Liu, Zhichao Zhang, Mingyang Ma

**Affiliations:** School of Electronic Engineering, Beijing University of Posts and Telecommunications, Beijing 100876, China; dingzk@bupt.edu.cn (Z.D.); liubingxun@bupt.edu.cn (B.L.); 2020110459@bupt.cn (Z.Z.); mingyangma@bupt.edu.cn (M.M.)

**Keywords:** multi-source sensor, scene classification, GNSS, data mining, CNN

## Abstract

Smartphones with integrated sensors play an important role in people’s lives, and in advanced multi-sensor fusion navigation systems, the use of individual sensor information is crucial. Because of the different environments, the weights of the sensors will be different, which will also affect the method and results of multi-source fusion positioning. Based on the multi-source data from smartphone sensors, this study explores five types of information—Global Navigation Satellite System (GNSS), Inertial Measurement Units (IMUs), cellular networks, optical sensors, and Wi-Fi sensors—characterizing the temporal, spatial, and mathematical statistical features of the data, and it constructs a multi-scale, multi-window, and context-connected scene sensing model to accurately detect the environmental scene in indoor, semi-indoor, outdoor, and semi-outdoor spaces, thus providing a good basis for multi-sensor positioning in a multi-sensor navigation system. Detecting environmental scenes provides an environmental positioning basis for multi-sensor fusion localization. This model is divided into four main parts: multi-sensor-based data mining, a multi-scale convolutional neural network (CNN), a bidirectional long short-term memory (BiLSTM) network combined with contextual information, and a meta-heuristic optimization algorithm.

## 1. Introduction

Smartphones are becoming an increasingly important part of people’s lives, accompanying us through 24 h a day [1,2]. With the construction of smart cities, smartphones play a vital role as device terminals, providing people with accurate positioning application services, such as seamless indoor and outdoor switching, tracking, smart shopping malls, smart car parks, and health monitoring. However, accurate, continuous, and reliable positioning services often cannot be achieved by a single source of positioning signals and always rely on a wide range of positioning methods, such as satellite positioning, Bluetooth positioning, Wi-Fi positioning, and various other methods. For example, to obtain more accurate, continuous, and reliable positioning services, these positioning methods are usually combined, resulting in multi-source fusion positioning methods. However, due to the different environments in which they are located, the types of sensors used can also vary significantly, making accurate scene judgment essential for determining reliable positioning [3,4,5].

In our daily lives, smartphones can often receive the main sources of signals—global positioning satellite signals, Bluetooth signals, Wi-Fi, light sensors, inertial navigation, and cellular networks—which enable the accurate identification of indoor and outdoor scenes. Smartphone users can invoke location services at any time and in any place, which could be in a playground at noon, an office building in the early hours of the morning, or a shopping mall on a Saturday or Sunday. In short, the varying times and places have a significant impact on location services [6,7,8].

According to the different types of sensors, scene classification algorithms can be divided into single-sensor scene classification algorithms and multi-sensor scene classification algorithms [9,10]. Among them, some studies have used single-sensor classification algorithms; single-sensor algorithms are simple in origin, and the classification model algorithms are relatively lightweight, but they are more restricted with poorer accuracy and poorer scene adaptation ability. SatProbe directly uses the number of visible satellites in the original satellite data to judge an IO scene, which contains 79 original satellite trajectories and 2595 detected points [11], and Ran et al. used machine learning to classify scenes for (2D) light detection and ranging (LiDAR) sensors on the FR079 public dataset as well as a laboratory dataset, both of which achieved better results [12]. Gao et al. extracted the number of visible satellites with carrier-to-noise ratios exceeding 25 dB and the sum of the signal strengths of all visible satellites with carrier-to-noise ratios exceeding 25 dB as features. Then, a machine learning model was used to infer the current scene type [13]. Wang et al. used the number of cells and base station signal strengths of cellular network base stations and exploited the variability of the cellular network distribution in indoor and outdoor scenes to achieve four types of scene classification [14]. Researchers have conducted a lot of research on single-sensor classification algorithms, but single sensors have limited access to information, and indoor and outdoor scene classification algorithms based on a single source are challenging.

In order to obtain higher-accuracy scene resolution, researchers have added more sensors and used multi-sensors to obtain more source information. SenseIO uses multiple sensors such as cellular networks, Wi-Fi, accelerometers, and lights in mobile phone sensors to build a SenseIO framework, and finally, it carries out detection on multiple application scenarios to achieve 92% accuracy [15]. Zhu et al. proposed a behavior-assisted hidden Markov model and classifier combination to achieve the best environmental detection accuracy of 94.22% on a complex building dataset [16]. Liu et al. proposed an indoor scene constraint-based localization method for accurate scene classification by fusing multiple camera, Wi-Fi, and inertial sensors [17]. IODetector utilizes lightweight sensors, including ambient light sensors, and cellular networks to distinguish between indoor and outdoor scenes using a joint strategy with fixed thresholds and Adaboost classifiers [18]. Anagnostopoulos et al. used the C4.5 algorithm for I0 detection, and features inputted to the model included feature information such as barometric pressure, light intensity, time, base station signals, magnetometer variance, etc. [19]. Li et al. used an algorithm based on Wi-Fi signal and light intensity fusion features to detect I0 scenes using AdaBoost and semi-conditional random fields [20].

The use of multi-sensor information can reduce the impact of sensor scene arrangement limitations and enriches the feature information so that a more accurate scene classification and recognition model can be obtained, while most multi-sensor feature scene classification models use deep learning or machine learning for the classification. At present, most of the research focuses on scene recognition accuracy and ignores the advantages that the data, temporal information, spatial information, and mathematical statistical information can bring, and it also ignores the role of indoor and outdoor scene discrimination for indoor and outdoor switching as well as the past based on fixed thresholds and special features. It is difficult to achieve good results in indoor and outdoor switching due to the complexity of signal changes when the scene is switched; it is difficult to detect scene switching changes correctly within a short period of time, and in addition to this, it is difficult to detect the scene switching changes correctly. Due to the complexity of signal changes during scene switching, it is difficult to correctly detect scene switching changes in a short time. In addition, the heterogeneity of equipment leads to signal fluctuations and thus causes feature drift, which makes the existing algorithms under-adapted in complex indoor and outdoor scenes. Therefore, this paper introduces a complex indoor and outdoor scene sensing algorithm based on spatio-temporal features.

The main contributions of this paper are as follows:

1. This paper proposes a complex indoor–outdoor scene sensing algorithm based on multiple signal sources, utilizing five types of sensors: global satellite navigation signals, cellular network signals, magnetometers, optical sensors, and Wi-Fi. The algorithm leverages both temporal and spatial features to handle complex scene detection.

2. The five sensors—global satellite navigation signals, cellular network signals, magnetometers, optical sensors, and Wi-Fi—are thoroughly characterized, and their data features are deeply mined. The data are analyzed from temporal, spatial, and mathematical/statistical perspectives to enrich and highlight the scene-specific features, enhancing the classification performance.

3. This paper introduces a scene classification network that uses multi-sensor data, comprising multi-scale CNN and BiLSTM architectures. This network effectively classifies data features and addresses the issue of long-term dependencies in neural networks.

4. The whale optimization algorithm is employed to automatically optimize key parameters of the neural network, including the initial learning rate, regularization parameters, and BiLSTM hidden layer settings. This method reduces the time required for manual parameter tuning and achieves improved performance.

The rest of the paper is organized as follows. Section 2 describes the data feature mining approach, data feature pre-processing, the proposed scene-aware classification model and evaluation metrics in this paper. Section 3 describes the dataset, which is followed by ablation experiments and a performance comparison with other methods. Section 4 discusses outlooks, and Section 5 concludes.

## 2. Materials and Methods

This section focuses on the characterization of data from GNSS, cellular network systems, optical sensors, magnetometers, and Wi-Fi sensors, all of which are commonly used in smartphones and are less affected by variations in terminal devices. These five sensors were chosen due to their widespread use and reliability. The temporal, spatial, and mathematical/statistical features of the data vary depending on whether the environment is indoor or outdoor, and thus, this paper emphasizes data mining for these five sensors to account for these differences.

**Figure 1:**
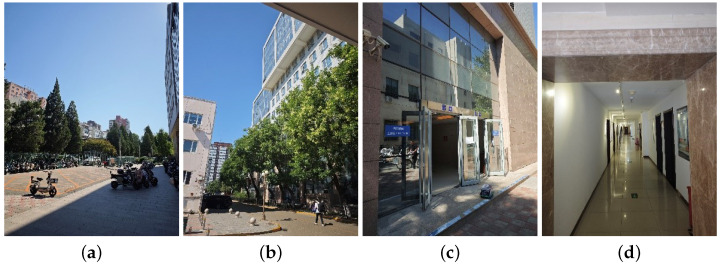
Four scene classifications: (**a**) outdoor, (**b**) semi-outdoor, (**c**) semi-indoor, and (**d**) indoor.

We divide the indoor–outdoor scenarios into four scenarios according to the scenarios, namely, open outdoor, semi-outdoor, semi-indoor, and indoor environments, as shown in Figure 1, in which open outdoor is defined as the positioning terminal not being surrounded by obstructions, semi-outdoor is defined as the location being partially obstructed by the building or other objects, semi-indoor is defined as the indoor environment near the window or the entrance/exit, and the indoor environment is defined as the location inside a building being partially obstructed by buildings or other objects.

### 2.1. Satellite Information Data Mining

**Figure 2:**
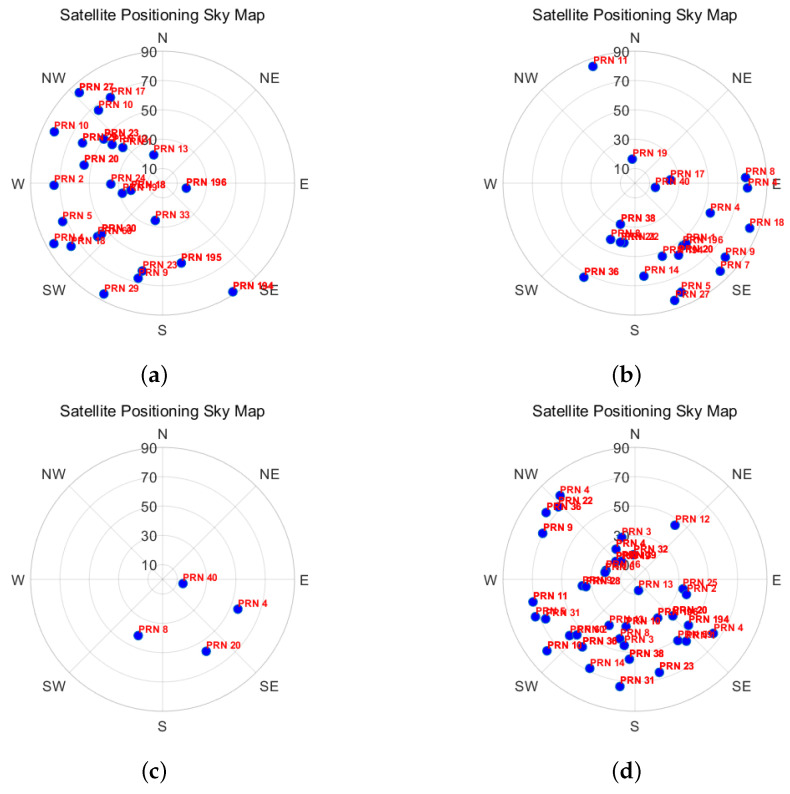
Satellite zenith view: (**a**) west indoor neighboring window, (**b**) south indoor neighbouring window, (**c**) indoor, and (**d**) open outdoor neighboring window.

Satellite azimuth and altitude angles indicate the position of a satellite in space. The azimuth angle is measured relative to true north, ranging from 0° to 360°, while the altitude angle ranges from 0° to 90°. Based on the distribution of visible satellite azimuth and altitude angles, it is possible to roughly infer areas of obstruction at the current location. Figure 2 illustrates this, showing the satellite zenith maps at various locations.

At the indoor west-side window, 90% of the visible satellites are distributed between 150° and 345°. Similarly, at the indoor south window, over 90% of visible satellites are located between 75° and 210°. In deeper indoor environments, only four visible satellites are detected, while in outdoor open areas, the satellites are more uniformly distributed. The altitude angle can also serve as a feature for scene classification.

Based on the above analysis, we divide the azimuth angle into 24 regions, each spanning 15 degrees, and construct a 24-dimensional feature vector to represent the satellite distribution. For each region, a value of 0 indicates no visible satellites, while a value of 1 indicates the presence of visible satellites. Additionally, the percentage of regions containing visible satellites relative to the total number of regions is also used as a feature input, which is represented by the following equation:

(1) $$P\_\mathrm{vis}\_\mathrm{sate}=\frac{\sum_{i=1}^{24}\;m_{i}}{24}$$

For precision analysis, we use the geometric dilution of precision (DOP) to assess how the spatial geometric distribution of observation satellites affects positioning accuracy. DOP is an indicator of position quality—higher DOP values indicate poor satellite geometry and lower precision, while smaller DOP values suggest a better satellite distribution and higher potential for precision.

The Position Dilution of Precision (PDOP) represents the three-dimensional position accuracy, the Horizontal Dilution of Precision (HDOP) measures the accuracy in the horizontal plane, and the Vertical Dilution of Precision (VDOP) focuses on vertical accuracy [21]. These factors describe the positioning accuracy in their respective dimensions, and their relationship is expressed as follows:

(2) $${\mathrm{GDOP}}^{2}={\mathrm{HDOP}}^{2}+{\mathrm{VDOP}}^{2}$$

**Figure 3:**
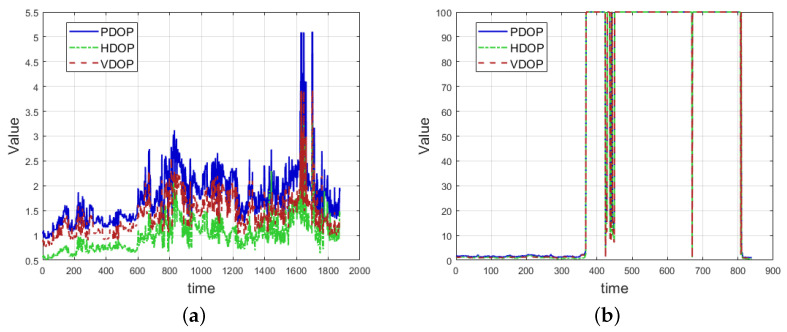
DOP change graph: (**a**) outdoor DOP change graph, and (**b**) indoor DOP change graph.

Researchers analyzed PDOP, HDOP, and VDOP statistics across various scenarios and derived their probability distributions, as shown in Figure 3. In Figure 3a, the graph represents the changes in accuracy factors from an open outdoor environment to a semi-outdoor environment. In open outdoor settings, the accuracy factors fluctuate less, but as the environment transitions to semi-outdoor, the fluctuations become more pronounced with 1127 calendar elements marking the transition.

Figure 3b shows the changes in accuracy factors as the environment shifts from semi-outdoor to semi-indoor and then to a deep indoor environment. At 352 calendar elements, the environment transitions to an indoor state, where the terminal remains in tracking mode without immediate changes in accuracy factors. However, sudden large fluctuations occur indoors, which is likely due to weak satellite signals in certain indoor areas.

Comparing the data from the open outdoor environment (calendar elements 0–1127) with the semi-outdoor environment (calendar elements 1128–3000), as shown in Table 1, Table 2 and Table 3, we analyzed the PDOP, HDOP, and VDOP values. It was observed that the minimum values in both outdoor and semi-outdoor environments were approximately the same, indicating that the minimum accuracy factor does not significantly differentiate these scenarios. However, the variance, peak, and mean values show clear differences and can be used as statistical features to distinguish between scenes.

In particular, the variance shows the most noticeable difference. In outdoor environments, the variance of the accuracy factor is generally low with values of 0.0060, 0.0015, and 0.0057, respectively. In contrast, in semi-outdoor environments, where objects like buildings cause obstructions, the variance is significantly higher with values of 0.2775, 0.1318, and 0.1644.

**Figure 4:**
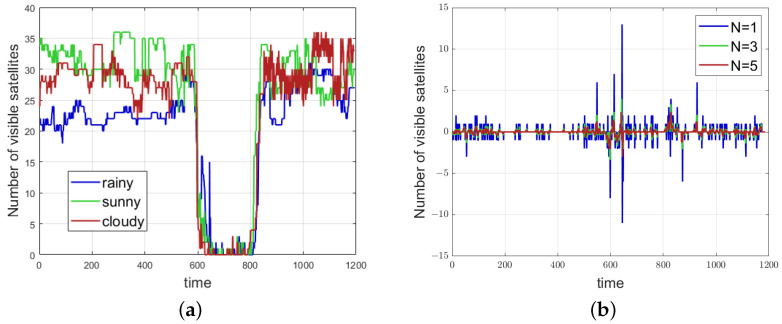
Visible satellite map: (**a**) variation in the number of visible satellites. (**b**) Variation in the rate of change of visible satellites in different windows.

The number of visible satellites can also serve as an important marker for scene classification. Changes in the number of visible satellites occur continuously, containing rich temporal information. Even at the same location, the number of visible satellites may vary under different weather conditions, as shown in Figure 4a. The figure depicts tests conducted under three weather conditions as the environment transitions from outdoor to indoor, then to semi-indoor, and back to outdoor.

On rainy days, the overall number of visible satellites is lower, likely due to the thicker cloud layers, which affect satellite signal reflection and refraction. Most of the time in outdoor environments, the number of visible satellites ranges between 20 and 25, but it fluctuates significantly when switching from outdoor to indoor. In deep indoor environments, the number of visible satellites drops to 2 or fewer. On cloudy days, the number of visible satellites mostly ranges from 25 to 30, which is likely due to cloud movement. This condition shows the greatest overall fluctuation. On sunny days, the number of visible satellites is the highest, typically ranging between 30 and 35.

The transitions between indoor and outdoor environments under the three different weather conditions reveal that the number of visible satellites does not immediately decrease upon moving indoors from outdoors. This is because the terminal maintains the tracking of satellite signals until the signal lock is lost, which occurs with a lag that is variable. Similarly, when moving outdoors from indoors, the number of visible satellites does not increase immediately. This delay is due to the time required for the terminal to capture, track, and process satellite signals, which involves solving the ephemeris. This process is influenced by both the terminal’s performance and environmental factors.

To minimize the influence of weather, environment, and equipment variability on the parameters, the number of visible satellites is processed using a time-series differencing method. The differencing formula is shown in the following equation, where D_num_sateN represents the number of difference features. $num_{k-N}$ indicates the number of visible satellites at moment k−N, and N refers to the number of calendar elements, which also determines the size of the sliding window.

When processing time-series data, the selection of the time window plays a critical role, as the features in the time series depend on the window size. As shown in Figure 4b, a small time window captures finer, localized features, while a larger time window reveals broader, global changes. This approach helps provide more useful information for scene classification by capturing multi-scale data. Consequently, time-series information is typically processed using multiple time windows to capture both small- and large-scale features, which are then used for scene classification.

(3) $$D\_\mathrm{num}\_\mathrm{sateN}=\frac{{\mathrm{num}}_{k}-{\mathrm{num}}_{k-N}}{N}$$

To evaluate the quality of a satellite signal, the most commonly used indicator is the carrier-to-noise ratio (CNR). The CNR is the ratio of the carrier signal power to the noise power spectral density, which is typically expressed in dBHz. This measure is crucial for assessing signal quality and the performance of the receiver. The CNR can vary significantly across different scenarios, such as indoor and outdoor environments, leading to different changes and results.

**Figure 5:**
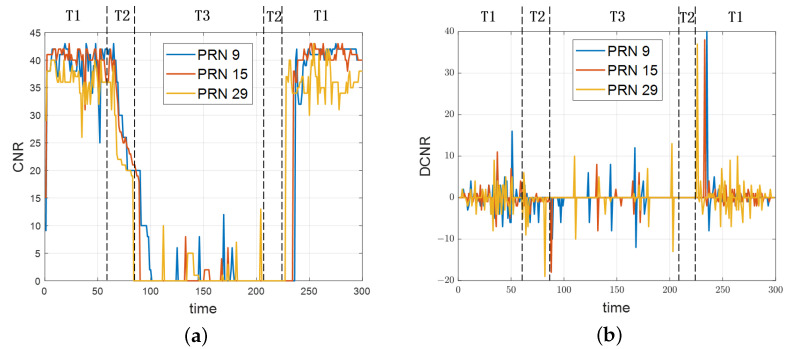
Satellite signal quality map: (**a**) CNR variation and (**b**) DCNR variation.

Figure 5 illustrates the variation in carrier-to-noise ratio (CNR) and the difference in CNR (DCNR) of three satellites during the indoor–outdoor state switching process. In the figure, T1, T2, and T3 represent the outdoor, semi-indoor, and indoor states, respectively. The analysis of Figure 5a reveals that when transitioning from the outdoor state to the indoor state, there is a noticeable time delay in the satellite CNR with a consistent downward trend observed over a certain period.

Figure 5b further indicates that during the transition from outdoor to indoor, the DCNR primarily consists of negative values for an extended duration. Conversely, in the transition from indoor to outdoor shown in Figure 5a, there is also a noticeable hysteresis effect with a steep increase in the CNR. This change results in several peaks in the DCNR, ranging from 30 to 40, as illustrated in Figure 5b.

Since different satellites exhibit varying trends, amplitudes, and timings in their CNR, it is essential to minimize the impact of these inconsistencies on scene classification. To address this, the algorithm analyzes the CNR and DCNR of all visible satellites to establish a comprehensive trend. By examining the peaks in DCNR changes, the algorithm can more accurately assess the transitions between indoor and outdoor states. Additionally, it considers the influence of sliding window size on this judgment. The definitions of the relevant formulas are as follows:

(4) $$f\left(x,x+N\right)=\mathrm{sign}\left(cnr_{i}^{x+N}-cnr_{i}^{x}\right)|i=0,1,\cdots ,n$$

(5) $$\;\mathrm{Peak}\;\left(x,x+N\right)=\max\left(cnr_{i}^{x+N}-cnr_{i}^{x}\right)|i=0,1,\cdots ,n$$

where $cnr_{i}^{x+N}$ denotes the CNR of the ith star at moment x, N denotes the sliding window size as well as the statistics of the percentage of ascending features, the percentage of descending features and the percentage of flat features within the sliding window. The formula can be expressed as

(6) $$P\left(\;\mathrm{down}\;\right)=\frac{n-\sum_{i=1}^{n}\mathrm{sign}\left(f\left(x,x+N\right)+1\right)}{n}$$

(7) $$P_{1}=\frac{\sum_{i=1}^{n}\mathrm{abs}\left(\mathrm{sign}\left(f\left(x,x+N\right)\right)\right)}{n}$$

(8) $$P\left(\;\mathrm{up}\;\right)=P_{1}-P\left(\;\mathrm{down}\;\right)$$

(9) P(hold)=1−P(up)−P(down)

### 2.2. Inertial Navigation Data Mining

Inertial navigation devices have become indispensable components in smartphones. The acceleration sensor, in particular, helps us determine the user’s state, allowing us to infer scene information from these state characteristics [22,23]. For example, if a user is in a moving vehicle, the likelihood of being in an outdoor environment can be inferred from the satellite visibility data, indicating the presence of outdoor scene information. Similarly, if the user is engaged in regular human movement, such as walking or running, there is also a high probability of being outdoors.

**Figure 6:**
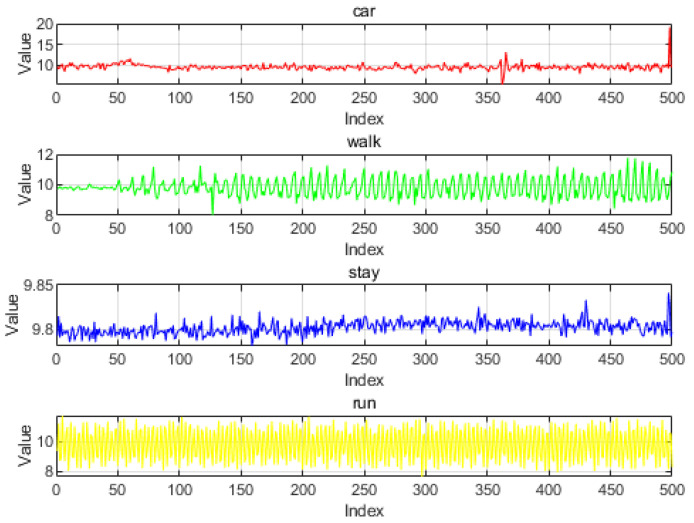
State of motion versus acceleration.

Figure 6 illustrates the relationship between the acceleration values from the smartphone’s inertial sensor and the corresponding motion states. We capture the raw information and then filter it to minimize noise and errors.

### 2.3. Wi-Fi AP Node Data Mining

Nowadays, Wi-Fi nodes are ubiquitous in society, and the AP (access point) nodes of Wi-Fi refer to wireless access points [24,25]. Generally, the number of AP nodes available outdoors is greater than those indoors, especially in dense urban areas, commercial streets, office buildings, campuses, and parks. In contrast, residential areas tend to have fewer AP nodes. When entering an urban indoor space, the presence of multiple walls can obstruct signals, leading to a generally lower number of accessible AP nodes compared to outdoor locations.

**Figure 7:**
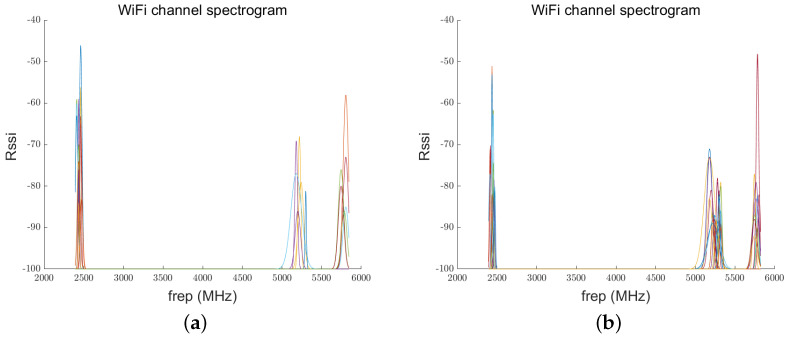
Wi-Fi channel spectrum scan: (**a**) indoor, (**b**) outdoor.

Figure 7a and Figure 7b depict the spectrum maps of indoor and outdoor Wi-Fi channels, respectively. The frequency points are primarily distributed in the 2.4 GHz, 5 GHz, and 6 GHz bands introduced by Wi-Fi 6E. It can be observed that nodes operating in the 2.4 GHz band typically exhibit stronger signal strength. In contrast, the signal strengths in the other frequency bands are generally lower. The 2.4 GHz band offers wider coverage and better penetration but is more susceptible to interference due to its narrower bandwidth.

The 5 GHz band provides a larger coverage area and more channels, resulting in higher data rates and reduced interference; however, it has smaller coverage and poorer penetration. The 6 GHz band offers even more channels and less interference, making it suitable for environments with high data demands, although its signal strength and coverage may be slightly reduced.

**Figure 8:**
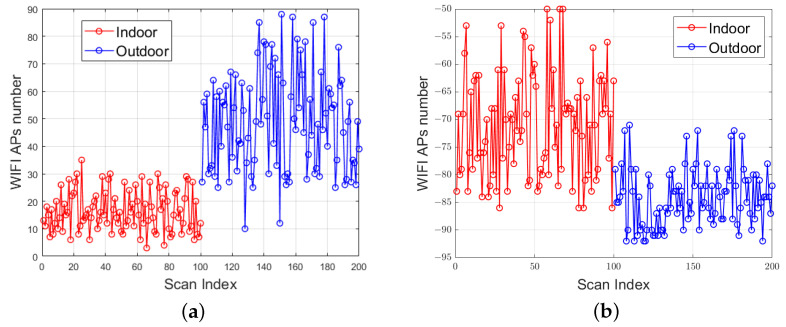
Visible AP distribution of Wi-Fi: (**a**) number distribution, (**b**) signal strength distribution.

Figure 8a presents the graph showing the number of Wi-Fi AP nodes. A total of 200 experiments were conducted, comprising 100 groups of indoor scanning results and 100 groups of outdoor scanning results across various environments, including office buildings, campuses, playgrounds, residential buildings, and shopping malls. The graph indicates that the number of indoor AP nodes typically ranges from 0 to 30, while the number of outdoor AP nodes generally falls between 25 and 90, demonstrating that outdoor AP nodes are typically more numerous than indoor nodes.

Figure 8b illustrates the Wi-Fi signal strength, which tends to fluctuate significantly indoors, with overall signal strengths ranging from −88 to −50 dBm. In contrast, outdoor signal strengths are lower, generally falling between −70 and −95 dBm. This difference can be attributed to the proximity of indoor Wi-Fi signals to the transmitting point, which experiences less obstruction. Conversely, outdoor signals are generally farther from the transmitting point but can receive a greater number of signals. Therefore, the number of Wi-Fi AP nodes and the average signal strength can also serve as important factors in scene classification.

### 2.4. Light Sensor Data Mining

As a crucial component of smartphones, light sensors enhance user experience and can also be utilized as part of scene-aware features [26,27]. Setting aside the effects of light sensor occlusion, we will analyze the relationship between light intensity and scene perception.

**Figure 9:**
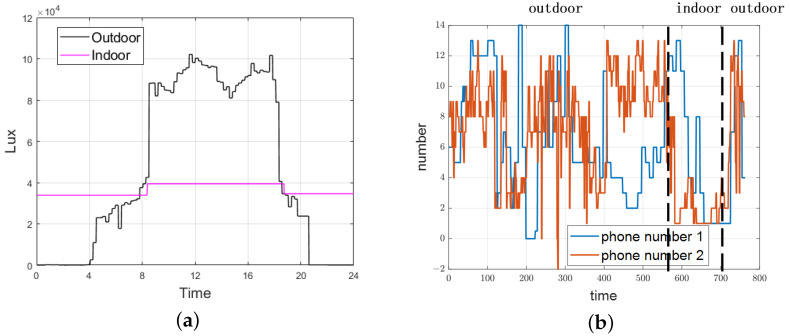
Variation of light sensors and cellular network sensors: (**a**) variation of indoor and outdoor light intensity over 24 h, (**b**) variation of the number of base stations receiving signals.

In our experiment, we placed sensors at indoor and outdoor nodes for 24 h and repeated the process 10 times to obtain average values, as illustrated in Figure 9a. The figure shows that outdoor light intensity varies significantly, peaking during the day at up to 100,000 Lux, while at night, it drops to only a few hundred Lux. This fluctuation is quite pronounced. During sunrise and sunset, the difference between indoor and outdoor light intensity becomes less distinct, making it challenging to differentiate between the two environments. However, at other times of the day, the distinction between indoor and outdoor light intensity is much clearer.

### 2.5. Cellular Network Data Mining

Cellular networks provide coverage for both indoor and outdoor areas, but the type of base stations used varies by environment. Suburban outdoor areas typically rely on macro-base stations, while urban outdoor areas often utilize micro-base stations. In contrast, indoor areas generally employ picocell base stations. These differences in base station design and transmit power significantly impact the coverage of cellular networks indoors and outdoors [28].

Figure 9b illustrates the number of cellular network signals received by two different devices in various indoor and outdoor environments. The overall fluctuation indoors is minimal, and the number of signals received is relatively low. This is primarily due to the limited number of pico-base stations and room substations available indoors. In contrast, outdoor areas experience greater fluctuations and a higher number of received signals, as they can access macro-base stations, micro-base stations, and a smaller number of pico-base stations.

From this analysis, we can deduce that the variance, peaks, valleys, and average of the signal strength, as well as the number of cellular base stations within a specified sliding window, can be used as key features for scene classification.

### 2.6. Complex Indoor and Outdoor Scene Classification Algorithm Based on Spatio-Temporal Features

In this paper, we propose a complex indoor and outdoor scene classification model based on spatio-temporal features. This model leverages data collected from cellular networks, satellite signals, Wi-Fi signals, light sensors, and inertial sensors found in smartphones. By performing spatio-temporal characterization and feature extraction on the collected information, we develop a network that integrates a multi-scale convolutional neural network (CNN) with a bidirectional long short-term memory (BiLSTM) network. The multi-scale CNN captures spatial features, while the BiLSTM further extracts spatio-temporal features from time series data.

**Figure 10:**
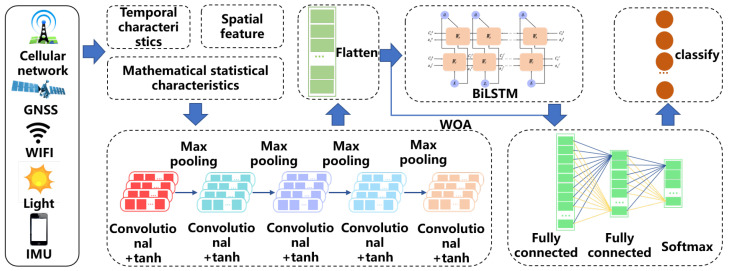
An algorithmic model for the classification of complex indoor and outdoor scenes based on spatio-temporal features.

The overall architecture of the algorithmic model is illustrated in Figure 10. In this model, features from the analyzed data are extracted through convolution, resulting in multiple feature sets that are then flattened. These features are passed through the BiLSTM network, and scene classification and identification are performed using a fully connected layer followed by a softmax activation function. Additionally, an intelligent optimization algorithm known as the whale optimization algorithm (WOA) is employed to determine the optimal parameter values. The data feature extraction process has been thoroughly described and standardized in the preceding sections.

To analyze the relationship between various sensor features and environmental characteristics, we introduced Pearson’s correlation coefficient to quantify the degree of linear correlation between two sensors [29]. This coefficient is expressed as follows:

(10) $$R=\frac{n\sum_{i=1}^{n}x_{i}y_{i}-\sum_{i=1}^{n}x_{i}\sum_{i=1}^{n}y_{i}}{\sqrt{\left(n\sum_{i=1}^{n}x_{i}^{2}-{\left(\sum_{i=1}^{n}x_{i}\right)}^{2}\right)\left(n\sum_{i=1}^{n}y_{i}^{2}-{\left(\sum_{i=1}^{n}y_{i}\right)}^{2}\right)}}$$

**Figure 11:**
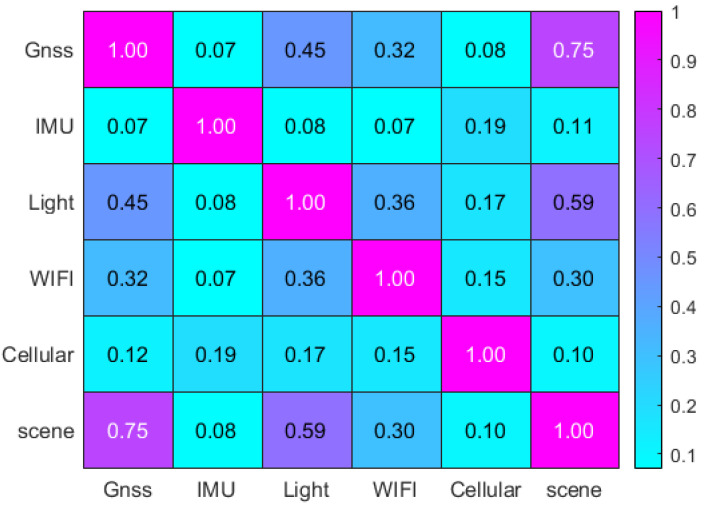
Pearson correlation feature map.

In the equation, $x_{i}$ and $y_{i}$ denote the first and second sensor variables, respectively, while n represents the number of samples. The calculation yields the correlation diagram of sensors and scene features, as shown in Figure 11. The correlation coefficients indicate that the relationships between the sensors are generally low with the GNSS sensors and light sensors exhibiting a stronger correlation. Furthermore, the GNSS sensors show the highest correlation with the scene, whereas the IMU sensors demonstrate the weakest correlation.

#### 2.6.1. Dual-Scale CNN Convolutional Networks

Convolutional networks are deep learning models designed for processing data with a grid topology. They automatically extract and classify features from data using convolutional, pooling, and fully connected layers. These networks have gained widespread application in fields such as image processing and computer vision. In recent years, researchers have achieved significant advancements in enhancing model efficiency, lightweighting, and task adaptability.

For instance, Bello proposed the ResNet-RS network, which optimizes the classical ResNet architecture. This enhancement improves performance on large-scale image classification tasks through better data augmentation, optimization techniques, and refined training processes [30]. Similarly, Liu et al. introduced ConvNeXt, which is a modernized version of the classical CNN. ConvNeXt adapts the original structure to close the performance gap with Vision Transformers while maintaining the efficiency characteristic of CNNs [31]. Additionally, Liu et al. developed MS-Net, which is a deep learning model aimed at improving prostate segmentation in MRI scans [32].

**Figure 12:**
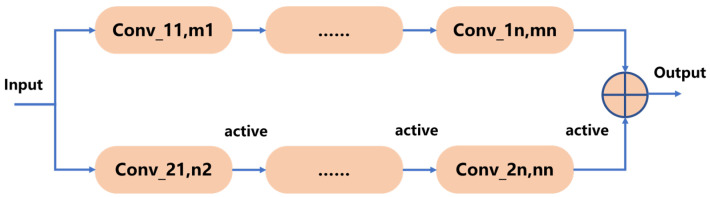
Schematic of a two-scale convolutional neural network.

In this paper, we employ a dual-scale convolutional network, as illustrated in Figure 12. This dual-scale neural network captures detailed information at different scales. Compared to traditional single-scale convolutional networks, the dual-scale approach allows for the extraction of feature information changes that may not be evident at a single scale. By integrating both local and global information, this method enhances the model’s generalization ability and supports more comprehensive decision making.

#### 2.6.2. BiLSTM Network

Although multi-scale neural networks effectively extract spatial features, their ability to capture temporal features is less satisfactory. To address this limitation, we incorporate a BiLSTM (bidirectional long short-term memory) network into the model for temporal feature extraction. LSTMs, or long short-term memory networks, consist of forget gates, input gates, and output gates, which work together as a memory unit. These gates dynamically regulate the storage and output of memory information at each time step.

In recent years, various LSTM variants have been proposed to enhance its capabilities [33]. For example, Qodim et al. introduced a spatio-temporal attention mechanism to improve video classification models. This approach utilizes an attention mechanism to weight important regions and time periods within video frames, enhancing the model’s ability to manage complex spatio-temporal dependencies in sequences. Additionally, they proposed a multi-scale LSTM model, which addresses sequential data across different time scales. This model enables LSTMs to capture features over varying time spans by integrating a multi-scale mechanism, making it suitable for processing temporal data with multiple cycles or hierarchical dependencies [34].

Moreover, Zhou et al. combined BiLSTM with graph neural networks (GNNs) for text classification tasks [35]. In this setup, the BiLSTM handles serialized features of the text, while the GNN further processes the graph structure, allowing for the better modeling of dependencies within the text.

**Figure 13:**
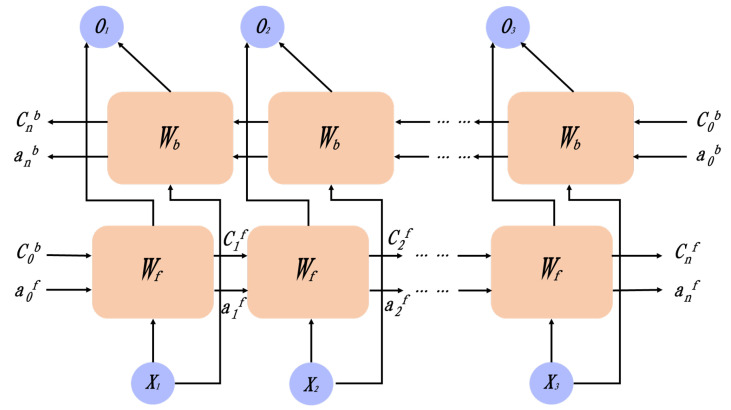
BiLSTM network structure diagram.

In this paper, we utilize a BiLSTM network, and its structure is illustrated in Figure 13.

This architecture significantly enhances the recognition of long-distance dependent patterns by simultaneously capturing the forward and backward temporal dependencies of data sequences. In this structure, the data first enter the bidirectional network, where the information flow to the hidden layer is precisely controlled through the coordinated tuning of two sets of parameters. Finally, the softmax layer is employed to effectively classify the extracted features. The theoretical expression for this process is as follows:

(11) $$\vec{h_{t}}=f\left(\vec{\omega }\cdot x_{t}+\vec{v}\cdot h_{t-1}+\vec{b}\right)$$

(12) $${\overset{\leftarrow }{h}}_{t}=f\left(\overset{\leftarrow }{\omega }\cdot x_{t}+\overset{\leftarrow }{v}\cdot h_{t-1}+\overset{\leftarrow }{b}\right)$$

(13) $$y_{t}=g\left(U\left[\vec{h_{t}},\overset{\leftarrow }{h_{t}}\right]+c\right)$$

where $\vec{h_{t}}$ is the BiLSTM forward layer output, ${\overset{\leftarrow }{h}}_{t}$ is the BiLSTM backward layer output, and $y_{t}$ is the hidden layer output. Since one-way LSTMs can only consider the influence of previous sequence data on the current data, they cannot incorporate feedback from later data to influence earlier judgments. This limitation prevents the integration of front and back sequences for comprehensive learning. In contrast, the model proposed in this paper possesses the capability to utilize contextual information, allowing it to make informed judgments by linking both past and future sequences.

#### 2.6.3. WOA Optimization Algorithm

The whale optimization algorithm (WOA) is an emerging meta-heuristic algorithm that explores the solution space by simulating the group behavior of humpback whales. It is known for its simplicity in implementation and strong global search capability [36]. The algorithm draws inspiration from the hunting techniques of humpback whales, which create bubble nets around their prey. During this hunting process, the whales swim in a spiral trajectory, gradually tightening their encirclement around the prey.

WOA models this hunting behavior through two primary mechanisms: encircling the prey and spiral motion.

In this context, we assume that the optimal solution corresponds to the location of the prey. As such, the whale will progressively approach the optimal solution. The algorithm formulates this behavior with the following equation:

(14) $$\vec{D}=|\vec{C}\cdot \vec{X^{*}}\left(t\right)-\vec{X}\left(t\right))|$$

(15) $$\vec{X}\left(t+1\right)=\vec{X^{*}}\left(t\right)-\vec{A}\cdot \vec{D}$$

(16) $$\vec{A}=2\vec{a}\cdot \vec{r}-\vec{a}$$

(17) $$\vec{C}=2\cdot \vec{r}$$

In the whale optimization algorithm, the position vector of the whale at the t-th generation is denoted as $\vec{X}\left(t\right)$, while $\vec{X}*\left(t\right)$ represents the current global optimal solution. Two dynamic vector coefficients, $\vec{A}$ and $\vec{C}$, are utilized in the algorithm. Coefficient A linearly decreases from 2 to 0 as the number of iterations progresses.

The value of $\vec{A}$ plays a crucial role in determining the whale’s movement dynamics. Specifically, when |A|< 1, the whale approaches the prey, while|A|> 1 prompts the whale to randomly select a new location, enhancing the diversity of the search process.

To simulate the spiral hunting behavior of the whale, a spiral motion model is introduced. This model allows the whale’s spiral trajectory to converge toward the prey, and the process is expressed as follows:

(18) $$\vec{X}\left(t+1\right)={\vec{D}}^{\prime }\cdot e^{bl}\cdot \cos\left(2\pi l\right)+\vec{X}*\left(t\right)$$

(19) $${\vec{D}}^{\prime }=\left|\vec{X}*\left(t\right)-\vec{X}\left(t\right)\right|$$

where b is a constant defining the shape of the spiral and l is a random number between [−1, 1]. The encircling prey and the updating mode are alternately updated, and the updating mode control can be expressed by the following equation:

(20) $$\vec{X}\left(t+1\right)=\left\{\begin{array}{cc} \vec{X}*\left(t\right)-\vec{A}\cdot \left|\vec{C}\cdot \vec{X}*\left(t\right)-\vec{X}\left(t\right)\right| & \;\mathrm{if}\;p<0.5\\ {\vec{D}}^{\prime }\cdot e^{bl}\cdot \cos\left(2\pi l\right)+\vec{X}*\left(t\right) & \;\mathrm{if}\;p\geq 0.5 \end{array}\right.$$

To reduce the chances of the algorithm getting stuck in a local optimum, an exploration mechanism is introduced in the early stages of iteration. During this exploration phase, the search will be conducted far from the current optimal solution to enhance global search capabilities. The formula for the exploration stage is as follows:

(21) $$\vec{X}\left(t+1\right)={\vec{X}}_{\mathrm{rand}\;}-\vec{A}\cdot \left|\vec{C}\cdot {\vec{X}}_{\mathrm{rand}\;}-\vec{X}\left(t\right)\right|$$

where ${\vec{X}}_{\mathrm{rand}\;}$ is a random solution in the current population. We use the WOA algorithm to train the initial learning rate, the regularization parameter and the number of BiLSTMs to improve the classification accuracy.

#### 2.6.4. Assessment of Indicators

The researchers used four metrics—accuracy, precision, F1 score, and recall—to evaluate the classification results of spatio-temporal scene recognition. The true positive (TP), false negative (FN), false positive (FP), and true negative (TN) are parameters obtained from the confusion matrix [37]. The accuracy rate represents the proportion of all samples that can be correctly predicted, and the equation is as follows:

(22) $$\;\mathrm{Accuracy}\;=\frac{\mathrm{TP}+\mathrm{TN}}{\mathrm{TP}+\mathrm{TN}+\mathrm{FP}+\mathrm{TN}}$$

Precision measures the proportion of positive samples that are correctly identified in the prediction results, and the equation is as follows:

(23) $$\;\mathrm{Precision}\;=\frac{\mathrm{TP}}{\mathrm{TP}+\mathrm{FP}}$$

Recall measures a classification model’s ability to recognize samples in the positive class (the class of interest). It is defined as the proportion of samples correctly identified as positive by the model out of all samples that are actually positive.

(24) $$\;\mathrm{Recall}\;=\frac{\mathrm{TP}}{\mathrm{TP}+\mathrm{FN}}$$

F1 combines two metrics, precision and recall, in the following equation:

(25) $$F1=2\times \frac{\;\mathrm{Precision}\;\times \;\mathrm{Recall}\;}{\;\mathrm{Precision}\;+\;\mathrm{Recall}\;}$$

The average value of F1 for all categories is called Macro-F1, and the equation is as follows:

(26) $$\mathrm{Macro}-F1=\frac{\sum_{1}^{n}{\left(F1\right)}_{n}}{n}$$

## 3. Results

To verify the effectiveness of the proposed classification model for complex indoor and outdoor scenes with spatio-temporal features, ten volunteers collected sensor data from smartphones used in their daily lives across multiple locations, including colleges and universities, shopping malls, downtown areas, and office buildings in cities such as Beijing, Guangzhou, and Hangzhou. We developed an application that can capture all sensor data information from the smartphones. The sampling rate for all data was set to 1 Hz, and the smartphones used were different models, which were all uniformly loaded with the application we created.

The volunteers followed designated routes inside and outside buildings while recording the current scene environment labels. The collected data were categorized based on these scene labels. The original data were divided into training, validation, and test datasets. The test dataset was specifically designed to assess the model’s generalization ability on new data. The details of the datasets are shown in Table 4.

### 3.1. Ablation Experiment

**Figure 14:**
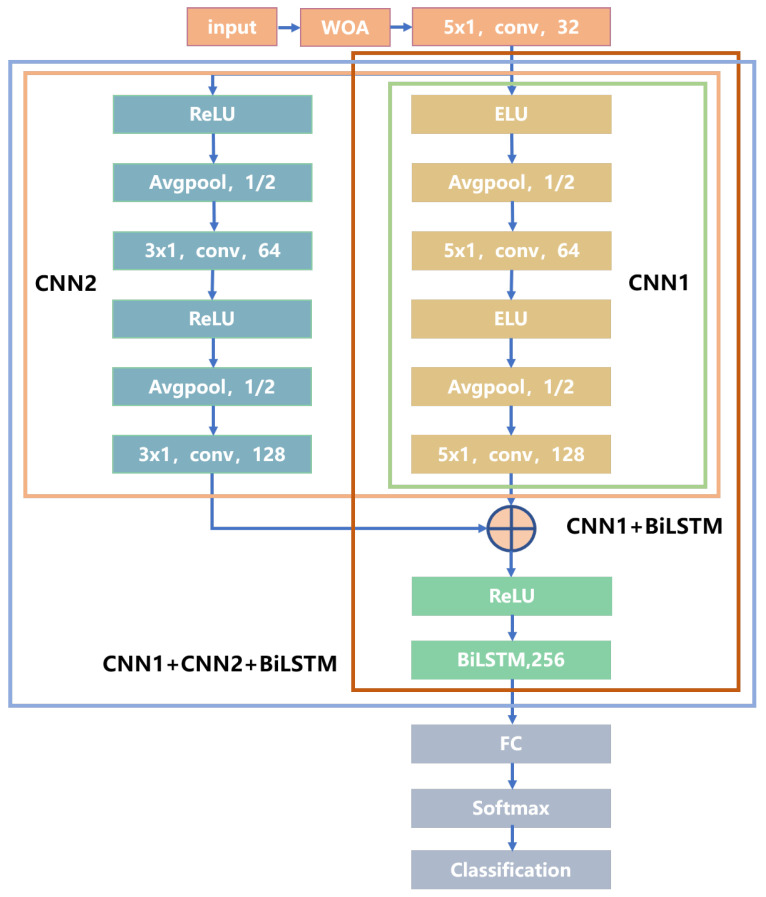
Structure of the ablation experiment.

In this section, ablation experiments are conducted to compare four different network configurations, as shown in Figure 14: CNN1, CNN1 + CNN2, CNN1 + BiLSTM, and CNN1 + CNN2 + BiLSTM. Each network is trained ten times to obtain the average results, which are presented in Table 5. The test results indicate that the classification accuracy of a single CNN network is 90.28%. In comparison, the feature accuracy of the dual-scale CNN network improves to 95.06% due to its enhanced feature extraction capabilities, combining both local and global features.

Furthermore, when comparing to the single CNN network, the accuracy of the BiLSTM network rises to 96.91%, benefiting from its superior ability to capture global and spatial features. By combining the dual-scale CNN and BiLSTM networks, the overall accuracy increases to 97.83%. For combined accuracy and recall, the F1 metric is employed, showing that the average F1 value for all categories rises to 0.9731 with the combination of the dual-scale CNN and BiLSTM networks. This represents an improvement compared to 0.8964 for the single CNN, 0.9481 for the dual-scale CNN (CNN1 + CNN2), and 0.9675 for the CNN1 + BiLSTM configuration, which also shows a corresponding increase.

**Figure 15:**
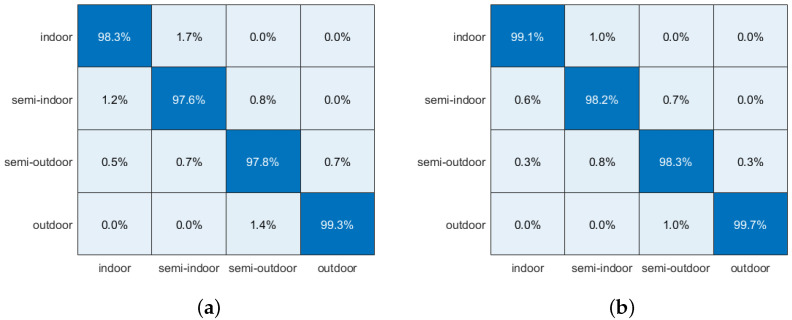
Confusion matrix: (**a**) confusion matrix before WOA optimization. (**b**) confusion matrix after WOA optimisation.

Figure 15a presents the confusion matrix for the combined classification of the dual-scale CNN network and the BiLSTM network. For the data labeled with the indoor category, 98.3% of the data are classified correctly, while 1.2% are misclassified as semi-indoor and 0.5% as semi-outdoor. This misclassification can be attributed to the similarity in features between the semi-indoor, semi-outdoor, and indoor scenes.

For the data labeled as semi-indoor, 97.6% are correctly classified while 1.7% are incorrectly identified as indoor and 0.7% as semi-outdoor. This indicates that the features of semi-indoor scenes are closer to those of indoor scenes, but there is still a notable chance of misclassifying them as semi-outdoor venues.

Similarly, for the data labeled as semi-outdoor, 97.8% are classified correctly, while 0.8% are misclassified as semi-indoor and 1.4% as outdoor. This suggests that although the semi-outdoor scene may experience some occlusion, the signal is not significantly affected in many environments. Lastly, 99.3% of the outdoor scenes are classified correctly, which is due to the more distinct characteristics of outdoor scenes, resulting in the highest classification accuracy among the four categories.

### 3.2. WOA Experiment

We chose a model that combines a dual-scale CNN network with BiLSTM for scene classification, which significantly enhances classification performance. However, the initial learning rate and regularization coefficients of the network model require optimization. To reduce the workload associated with manual parameter tuning and improve scene classification accuracy, we employ the WOA algorithm for automatic parameter adjustment. The WOA has been described in detail above. We utilize the Iterative Training Accuracy (ITA) to monitor the changes in accuracy throughout the training process, reflecting the optimization effects of WOA. The formula for ITA is as follows:

(27) $${\mathrm{ITC}}_{i}=1-{\left({\mathrm{Accuracy}}_{\;\mathrm{train}}\right)}_{i}$$

where i is the number of iterations, ${\mathrm{Accuracy}\;}_{\mathrm{train}}$ is the training accuracy, which according to the value of ITC can determine whether the WOA reaches the optimal solution. We select the CNN1 + CNN2 + BiLSTM network, which showed the best performance in the previous section, and further optimize it using the WOA algorithm. The WOA algorithm iteratively optimizes the parameters with an initial learning rate of 0.0116 and an L2 regularization parameter of 0.0012. The confusion matrix after WOA optimization is shown in Figure 15b. The right-most column of Table 6 presents the results of the WOA algorithm optimization for the CNN1 + CNN2 + BiLSTM network, which improves accuracy to 98.83% and increases the average F1 value to 0.988 compared to the previous results of the CNN1 + CNN2 + BiLSTM network. Additionally, the correct classification rates for all four types of scene classifications have increased. Therefore, the WOA algorithm not only reduces the time required for manual parameter adjustment but also enhances the accuracy of scene classification.

### 3.3. Experimental Comparison

**Figure 16:**
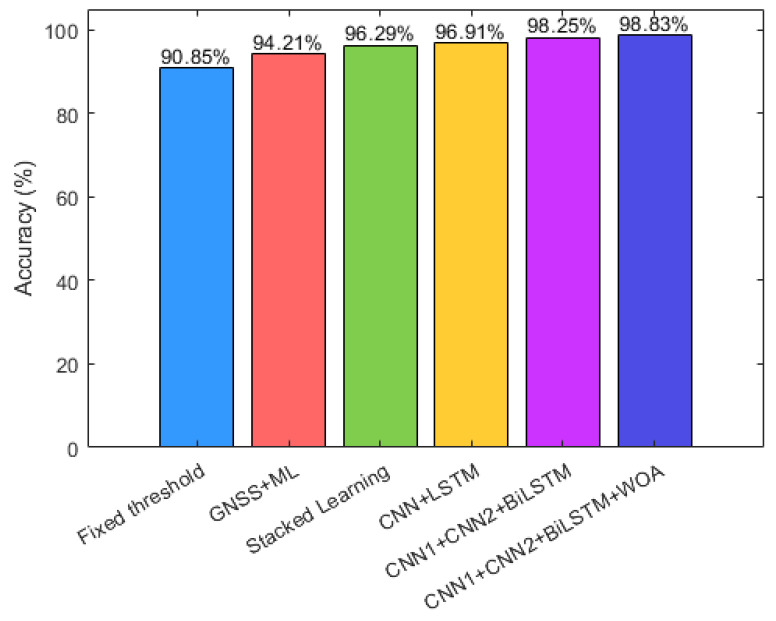
Comparison of the accuracy of different models.

Several advanced algorithms have been selected for comparison. The first algorithm is based on the number of visible satellites. This method uses a fixed threshold for the number of visible satellites to make judgments. While it is simple, it has a high error rate and relies on a single source of information. The second algorithm also considers the number of visible satellites along with the carrier-to-noise ratio (CNR) and other information, classifying the data using machine learning. This approach utilizes various signal features from a single source and incorporates machine learning for more flexible judgments. However, it can be easily affected by the sensor state, leading to misjudgments. The third algorithm employs stacked integrated learning, which enhances scene classification ability, but its feature mining capabilities are insufficient, resulting in poor generalization. The fourth method combines convolutional neural networks (CNN) with long short-term memory (LSTM) networks for scene classification. The fifth method is the algorithm proposed in this paper. Both datasets were run ten times to calculate average values, and the results for the fixed threshold method are shown in Figure 16.

The first method uses a fixed threshold approach, achieving a scene recognition accuracy of 90.85%. Although its threshold setting is straightforward, the scene-switching thresholds vary between cities, resulting in lower scene classification accuracy. The second method utilizes the number of visible satellites, carrier-to-noise ratio (CNR), and other information, classifying the data with machine learning to achieve an accuracy of 94.21%. This improvement over the first method is due to both the increased dimensionality of satellite data features and the enhanced accuracy of the machine learning model. However, it still relies on a single sensor signal, leading to weaker anti-interference capability.

The third method employs stacked integrated learning, increasing scene classification accuracy to 96.29%. This method not only extracts features from a single satellite signal but also leverages data from multiple sensors, resulting in richer features. However, it falls short in mining signal features adequately, leading to weaker generalization ability. The fourth method achieves an accuracy of 96.91% by using a CNN + LSTM network, which can extract scene features more effectively, yet it still struggles with correlating signal features and scene features.

The fifth method combines a dual-scale CNN network with a BiLSTM network, reaching an accuracy of 98.25%. This approach excels at extracting features both locally and globally as well as from a sequential perspective, incorporating temporal, spatial, and mathematical statistical features from multiple sensors. It closely associates these features with scene labels, surpassing the accuracy of the previous four methods. Finally, the sixth method applies parameter optimization using the whale optimization algorithm (WOA) to the fifth method, achieving an accuracy of 98.83%, which is the highest among all the methods evaluated.

**Figure 17:**
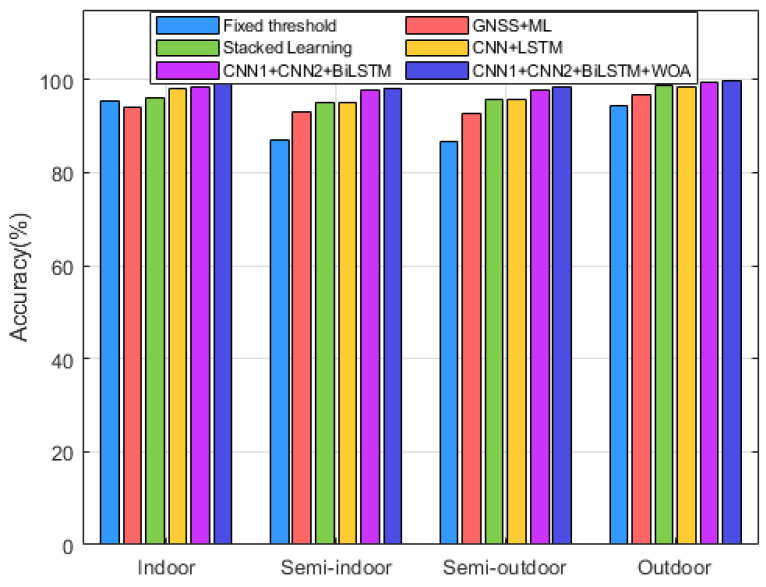
Comparison of accuracy in different scenarios.

Figure 17 presents a histogram showing the accuracy of the six methods across four scenarios, clearly illustrating how these algorithms classify different situations. The first method, based on fixed thresholds, achieves accuracy rates of 95.3% and 94.3% for indoor and outdoor scenarios, respectively. However, its performance drops significantly for semi-indoor and semi-outdoor scenarios with accuracies of only 87.1% and 86.7%. This decline can be attributed to the fixed threshold approach’s limited recognition ability in semi-indoor and semi-outdoor scenarios, where satellite signal features exhibit less distinct variations. In contrast, the other networks that utilize training models demonstrate improved accuracy across all four scenarios.

For semi-indoor and semi-outdoor scenes, the accuracy of the CNN + BiLSTM network is significantly higher than that of the other algorithms. This indicates that this network’s ability to extract temporal and spatial information during state transitions is markedly superior to that of other methods, such as the stacked model. In the case of the latter four methods, the use of multi-source feature data results in significantly better accuracy across all four scenes compared to scene classification algorithms that rely on single-source information. This improvement is attributed to the inherent homogeneity of single sensor information; data from different sensors can characterize scene features from multiple perspectives.

## 4. Discussion

Compared with traditional scene classification algorithms, such as fixed threshold methods and stacked learning, this algorithm fully integrates temporal, spatial, and mathematical statistical features. It combines scene features with signal features, constructing a model that merges a dual-scale CNN network with a BiLSTM network optimized by the WOA algorithm. Experiments conducted on multi-source scene sensing in smartphones demonstrate that this algorithm exhibits superior performance and higher accuracy rates.

The current algorithm can be applied to multi-source fusion localization in smartphones, providing enhanced a priori information and scene data for multi-sensor fusion localization, improving the accuracy of sensor fusion and switching. Additionally, the accuracy and recognition speed of this model in scene switching zones still hold significant potential for future research directions.

In future research on algorithms for classifying complex indoor and outdoor scenes based on spatio-temporal features, we should continue to deepen the fusion and analysis of multi-source sensor data. Additionally, efforts should be made to enhance the robustness and real-time performance of the model in response to changes in complex environments. This research should focus on optimizing the structure of deep learning models, improving feature extraction capabilities, and exploring more efficient algorithm optimization techniques.

At the same time, emphasis should be placed on the cross-scene applicability and interpretability of the model with the goal of developing a more accurate, reliable, and universal indoor–outdoor scene classification system. This system should be sensitive enough to classify scene feature data and rapidly aggregate the features that detect scene changes into a switching-awareness model, facilitating faster and more accurate indoor–outdoor fusion switching. Regarding the seamless integration of indoor and outdoor environments, it is essential to ensure that an accurate perception of both environments enhances the degree of seamlessness. We can improve the network model to achieve faster scene recognition and enable data prediction based on changes in data features, allowing for quicker and more accurate judgments regarding seamless scene switching.

## 5. Conclusions

In this paper, we propose a novel scene perception model that leverages multi-source data from smartphones to explore the relationship between scene environments and information features from three perspectives: time, space, and mathematical statistical models. This model deep mines five information sources—satellite signals, cellular networks, Wi-Fi, IMU, and light intensity—and integrates a whale optimization algorithm (WOA)-optimized dual-scale CNN network with a BiLSTM network.

First, we conduct ablation experiments using a dataset collected from multiple cities, devices, and scenes. The proposed algorithm achieves an accuracy of 98.83%, demonstrating both a strong performance and generalization ability on the test set. Finally, comparisons with other existing advanced algorithms reveal that our proposed model exhibits a higher accuracy rate and optimal performance across four scenarios. This enhanced accuracy can better support scene perception and recognition for smartphones, providing a robust environment-aware foundation for plug-and-play multi-source fusion intelligent localization.

## Figures and Tables

**Table 1:** PDOP outdoor vs. semi-outdoor statistics comparison.

Form	Variance	Peak	Min	Mean
Outdoor	0.0060	1.3700	0.9500	1.1140
Semi-outdoor	0.2775	5.1000	0.9300	1.7588

**Table 2:** HDOP outdoor vs. semi-outdoor statistics comparison.

Form	Variance	Peak	Min	Mean
Outdoor	0.0015	0.7600	0.5300	0.5912
Semi-outdoor	0.1318	3.2700	0.5000	1.0238

**Table 3:** VDOP outdoor vs. semi-outdoor statistics comparison.

Form	Variance	Peak	Min	Mean
Outdoor	0.0057	1.1600	0.7700	0.9434
Semi-outdoor	0.1644	3.9100	0.7700	1.4238

**Table 4:** Distribution of datasets.

Dataset	Total Number of Scenarios	Data Volume	Municipalities	Scenario Categories Involved
Training set	217	105,164	Beijing, Guangzhou, Hangzhou	Indoor, semi-indoor, outdoor, semi-outdoor
Validation set	34	29,178	Beijing, Guangzhou, Hangzhou	Indoor, semi-indoor, outdoor, semi-outdoor
Test set	71	48,536	Beijing, Shanghai	Indoor, semi-indoor, outdoor, semi-outdoor

**Table 5:** Results of ablation experiments.

	CNN1	CNN1 + CNN2	CNN1 + BiLSTM	CNN1 + CNN2 + BiLSTM
Accuracy (%)	90.28	95.06	96.91	98.25
Macro-F1	0.8964	0.9481	0.9675	0.9810

**Table 6:** Results of WOA experiment.

	CNN1 + CNN2 + BiLSTM	CNN1 + CNN2 + BiLSTM + WOA
Accuracy (%)	98.25	98.83
Macro-F1	0.9810	0.9880

## Data Availability

The data that support the findings of this study are available from the corresponding author upon reasonable request.

## Abbreviations

The following abbreviations are used in this manuscript:

GNSS	Global Navigation Satellite System
IMU	Inertial Measurement Unit
CNN	convolutional neural networks
BiLSTM	bidirectional long short-term memory
LiDAR	light detection and ranging
DOP	dilution of precision
HDOP	Horizontal Dilution of Precision
VDOP	Vertical Dilution of Precision
PDOP	Position Dilution of Precision
CNR	carrier-to-noise ratio
DCNR	difference in carrier-to-noise ratio
GNN	graph neural networks
WOA	whale optimization algorithm
TP	true positive
FN	false negative
FP	false positive
TN	true negative
